# The Public Services

**Published:** 1911-09-02

**Authors:** 


### The Public Services.

### THE NAVY MEDICAL DEPARTMENT.

This Service is only open to the unmarried registered graduate of European birth between 21 and 28 years of age. He must pass an entrance examination and gain at least one-third of the possible marks, and he must satisfy the physical requirements of the Medical Board.

The examination will consist of two parts, as follows: (i) compulsory subjects, and (ii) voluntary subjects. The former are (*a*) medicine, materia medica, therapeutics, and general hygiene; (*b*) surgery and surgical anatomy; (*c*) pathology. The attention of candidates is especially drawn to the importance of the section of Operative Surgery. No candidate will be considered eligible who has not obtained at least one third of the maximum marks in each of the above compulsory subjects. The voluntary subjects are French and German. The knowledge of modern languages being considered of great importance by the authorities, all intending competitors are urged to qualify in French and German.

Successful candidates immediately after passing the examination will receive commissions as surgeons in the Royal Navy, and undergo a course of practical instruction in naval hygiene, the diseases of warm climates, bacteriology, surgery of gunshot wounds, etc., radiography, and the photography in connection with it, at Haslar Hospital.

Every medical officer will be required to undergo a post-graduate course of three months' duration at a metropolitan hospital once in every eight years (should the exigencies of the service permit), and this as far as possible during his surgeon's, staff surgeon's, and fleet surgeon's period of service. While carrying out this course the medical officer will be borne on a ship's books for full pay, and will be granted lodging and provision allowances, and travelling expenses as for service under the regulations to and from his home or port; the fees for each course (not exceeding £25) will be paid by the Admiralty on the production of vouchers at the end of the course. The medical officer will be required to produce separate certificates of efficient attendance in the following: I. The medical and surgical practice of the hospital; II. A course of operative surgery on the dead body; III. A course of bacteriology; IV. A course of ophthalmic surgery, particular attention being paid to the diagnosis of errors of refraction; V. A practical course of skiagraphy.

### THE ROYAL ARMY MEDICAL CORPS.

A candidate for a commission in the Royal Army Medical Corps must be 21 years and not over 28 years of age, and must possess a registered qualification to practise. He must pass an entrance examination, and, having gained a place and having satisfied the Medical Board of his physical fitness, he must undergo two months' instruction in hygiene and bacteriology, and afterwards proceed to Aldershot for instruction in the technical duties of the corps.

The entrance examination is held twice a year in London, usually in January and July, and an entrance fee of £1 is charged for admission to it. It lasts about four days. The examination is of a clinical and practical character, partly written and partly oral. It consists of examination and report upon a medical case and commentary upon a case in medicine; clinical medical cases and *viva voce* on medical pathology; examination and report upon a surgical case and commentary upon a case in surgery; clinical surgery and pathology (including diseases of the eye), and operative surgery, bandaging, surgical instruments and appliances.

### THE INDIAN MEDICAL SERVICE.

Candidates must be natural-born subjects of His Majesty, of European or East Indian descent, between 21 and 28 years of age at the date of the examination, of sound bodily health, and in the opinion of the Secretary of State for India in Council in all respects suitable to hold commissions in the Indian Medical Service. They may be married or unmarried. They must possess, under the Medical Acts in force at the time of their appointment, a registrable qualification to practise both medicine and surgery in Great Britain and Ireland.

The physical fitness of each candidate will be determined by a Board of Medical Officers, who are required to certify that his vision is sufficiently good to enable him to pass the tests laid down by the Regulations. The candidate will be examined by the Examining Board in the following subjects: (1) medicine, including therapeutics; (2) surgery, including diseases of the eye; (3) applied anatomy and physiology; (4) pathology and bacteriology; (5) midwifery and diseases of women and children; (6) materia medica, pharmacology, and toxicology. The examination in medicine and surgery will be in part practical, and will include operations on the dead body, the application of surgical apparatus, and the examination of medical and surgical patients at the bedside. No syllabus is issued in the subjects the examination which will be conducted so as to test the general knowledge of the candidate in these subjects. No candidate shall be considered eligible who shall not have obtained at least one-third of the marks obtainable in each of the above subjects and one-half of the aggregate marks for all the subjects.

After passing examination, the successful candidates will be required to attend a course of practical instruction at the Army Medical School and elsewhere, as may be decided, in: (1) hygiene; (2) military and tropical medicine; (3) military surgery; (4) pathology of diseases and injuries incidental to military and tropical service. This course will be of not less than four months' duration, and at its conclusion candidates will be required to pass an examination in the subjects taught therein.

### THE COLONIAL MEDICAL SERVICE.

Full details regarding this service and the advantages it offers to newly qualified men will be found in the special article "The Colonial Medical Service," on page 569 of the present issue. Further details may be obtained on application to the Colonial Office.

